# Confirming the Environmental Concerns of Community Members Utilizing Participatory-Based Research in the Houston Neighborhood of Manchester

**DOI:** 10.3390/ijerph13090839

**Published:** 2016-08-23

**Authors:** Garett Sansom, Philip Berke, Thomas McDonald, Eva Shipp, Jennifer Horney

**Affiliations:** 1 Health Science Center School of Public Health, Texas A&M University, College Station, TX 78665, USA; tmcdonald@sph.tamhsc.edu (T.M.); eshipp@sph.tamhsc.edu (E.S.); horney@sph.tamhsc.edu (J.H.); 2 Department of Landscape Architecture, Texas A&M University, College Station, TX 77840, USA; pberke@arch.tamu.edu

**Keywords:** environmental justice, community engagement, participatory-based research, environmental equity, disaster preparedness, water quality, water sampling

## Abstract

In the last few decades, there has been an increase in community-based participatory research being conducted within the United States. Recent research has demonstrated that working with local community organizations, interest groups, and individuals can assist in the creation of, and sustainability in, health initiatives, adoption of emergency protocols, and potentially improve health outcomes for at-risk populations. However little research has assessed if communal concerns over environmental contaminants would be confirmed through environmental research. This cross-sectional study collected survey data and performed surface water analysis for heavy metals in a small neighborhood in Houston, TX, which is characterized by industrial sites, unimproved infrastructure, nuisance flooding, and poor air quality. Surveys were completed with 109 residents of the Manchester neighborhood. Water samples were taken from thirty zones within the neighborhood and assessed for arsenic (As), barium (Ba), cadmium (Cd), chromium (Cr), lead (Pb), selenium (Se), silver (Ag), and mercury (Hg). Survey results showed that the vast majority of all respondents were concerned over proximity to industry and waste facilities, as well as exposure to standing surface water. Barium was discovered in every sample and many of the zones showed alarming levels of certain metals. For example, one zone, two blocks from a public park, showed levels of arsenic at 180 (μg/L), barium at 3296 (μg/L), chromium at 363 (μg/L), lead at 1448 (μg/L), and mercury at 10 (μg/L). These findings support the hypothesis that neighborhood members are aware of the issues affecting their community and can offer researchers valuable assistance in every stage of study design and execution.

## 1. Introduction

Recent research has demonstrated many potential benefits of engaging community members and interest groups in the conduct of research and the development of interventions to improve outcomes [1,2,3,4]. Traditional approaches to the public assessment of environmental hazards typically have not included local residents in the identification of areas of concern, as they were often viewed as lacking the required expertise to adequately assess risk. The recent resurgence of community engagement has suggested that these approaches did not produce the same outcomes as studies that engage local knowledge in every phase of research [5]. While systematic reviews have confirmed local and governmental action have been improved with participation of local citizens and interest groups [6], little research has focused on how accurately the problems identified by the community are mirrored in research investigating the concerns of local citizens. This study uses the neighborhood of Manchester within Houston, TX, as a case study to identify the benefits of using local knowledge to focus on environmental hazard research.

Across multiple topics, including healthcare, clinical care, and applying research in novel environments, recent research has shown that interventions that utilize local residents and interest groups have greater success with enacting change in communities [7,8]. In a review of the impact of participatory-based research studies, Cashman et al. (2008) [9] examined the results of a study with Latino men in rural North Carolina where members were involved in every phase of data analyses and interpretation. This health-focused coalition concluded with the creation of an HIV and sexual transmitted diseases (STD) prevention initiative and a capacity building group. Due to the input from the community, specific programs were created and maintained longer than expected from most health intervention education programs.

Another intervention conducted by Bluthenthal et al. (2006) [3] targeted African Americans and Latinos in Los Angeles, CA, to reduce rates of depressive disorders, as well as educate the community on opportunities for assistance and address the gap between minority and majority populations. This program, with the help of local activist organizations, conducted an initial kickoff event that lead to the identification of many areas of concern, as well as identifying local members who could provide assistance. This pilot study developed into a program that is continuing to address the needs of the community through local services.

Hazard planning and mitigation has also been shown to benefit from broad communal participation in planning. For example, in a study conducted by Stevens et al. (2010) [10], 65 locations throughout the U.S. that experience high levels of natural hazards showed a statistically significant (*p* < 0.05) correlation between participation levels and implementation of hazard mitigation techniques. Engaging socially vulnerable groups, in every stage of hazard planning and mitigation, is particularly important as these groups face additional hurdles of discrimination, class inequality, and view “outside” interventions with higher levels of distrust and suspicion compared to majority groups [11,12]. Another study that examined six disadvantaged communities within the 2003 Hurricane Isabel impact zone under the Emergency Preparedness Demonstration project found that working with community members was invaluable. Researchers Berke et al. concluded that evidence suggest that “people have the power to build resiliency of their communities from within” [13].

This case study explores the benefits of community engagement in a community survey on perceptions related to the health impacts of environmental risk. In this project, community engagement techniques were used to better support the expertise of researchers with the local knowledge of community members and organizations already established in the community. This case study uses the concerns of the community, through outreach and neighborhood surveying, to determine if their concerns are confirmed through environmental and population research.

## 2. Materials and Methods 

### 2.1. Study Location and Population

Manchester, Texas, is a small neighborhood in eastern Houston located on the Houston Ship Channel. Manchester is primarily Non-White Hispanic and has endured numerous issues with flooding [14], air pollution [15], and health concerns [16]. Houston Ship Channel communities are at particularly high risk of impacts from the nexus of exposure to hazardous substances and natural disasters. For example, within one mile of the Manchester neighborhood, there are 21 facilities that report to the EPA’s Toxic Release Inventory: 11 large quantity generators of hazardous waste, four facilities that treat, store, or dispose of hazardous wastes, nine major dischargers of air pollution, and eight major storm water discharging facilities [17]. The area is also highly vulnerable to the impacts of natural disasters, both socially and physically. Houston has been divided into 88 separate areas called “Super Neighborhoods”, these neighborhoods include a council that serves as a forum for community concerns. Manchester is within Super Neighborhood 65 as part of the Harrisburg/Manchester Park neighborhood. The population of the Manchester Super Neighborhood is 98% minority, with a median income that is one-third less than the City of Houston overall. Only six percent of residents have obtained a Bachelor’s degree [18]. Floodplains along the Sims Bayou have increased by 15 percent since 1980, due to increases in development and impervious cover, like concrete and asphalt, while expected sea-level rise, due to climate change, could expose another 35,000 residents in Ship Channel neighborhoods to flooding [19].

### 2.2. Community Meeting

This study was a part of the Resilience and Climate Change Cooperative Project (RCCCP) which is a multi-year collaborative research and engagement program at Texas A&M University [20]. The goal of the RCCCP is to create a fundamentally different way to identify and address critical disaster resiliency and climate change challenges that threaten coastal cities. As a part of this broader RCCCP group, a community engagement meeting was held with local interest groups and individuals within the Harrisburg/Manchester neighborhood during the spring of 2015. Our research initiative was invited to collaborate with the community by the Texas Environmental Justice Advocacy Services (TEJAS) [21], the Green Ambassadors from Houston’s Furr High School [22], and in attendance were interested residents of the Harrisburg/Manchester neighborhood.

A brief presentation was given to the attendees on potential research and community activities planned in the coming months. Following the presentation those who were present were invited to fill out a short feedback form asking for suggestions as well as to provide information on what they thought their community suffered from the most. An opportunity to speak about these issues was also allowed and a member of the RCCCP wrote down the topics and concerns that were raised. This community meeting recorded concerns on issues related to health, the environment, education, and infrastructure. Some of these responses guided the direction of this research.

### 2.3. Survey Sample

Due to the relatively compact geography of the Manchester neighborhood, a complete census was attempted. Trained survey teams used paper surveys and walked every public road and passed every home within the borders of Manchester during two data collection days in December 2015. Homes that were completely fenced off, abandoned, or were deemed unsafe by the interview team were the only homes not approached during the canvasing.

Community partners that were already engaged from the previous meetings and other community engagement and research projects of the RCCCP assisted with survey data collection to help increase response rates. Specifically, the Green Ambassadors from Houston’s Furr High School and the EpiAssist program at the Texas A&M University Health Science Center School of Public Health [23] volunteered to help collect survey data. Teams were assembled that consisted of two or three individuals; each team included a graduate student from the EpiAssist program and at least one Spanish speaker. Written training materials as well as in-person sessions were held to adequately prepare the interviewers for collecting environmental health and perceptions data.

The survey consisted of 24 questions that included demographic information (gender, race, and age) and language proficiency (can anyone in the household speak English well). It also asked questions about the participant’s current view about environmental issues that may or may not be impacting their community. These questions included issues of pollution, natural disasters, and infrastructure. The participants were asked if they thought their community had issues with any of the following exposures: living near too many waste facilities, living near too many industrial buildings, living in buildings that need repair, exposure to standing water, and having poor road infrastructure as dictated by potholes. Each response had a binary outcome (yes or no). The survey offered respondents the opportunity to include concerns not mentioned in the survey. The survey and accompanying informed consent materials were approved by the Texas A&M University Institutional Review Board (#15-0648D).

### 2.4. Surface Water Sampling

**Figure 1:**
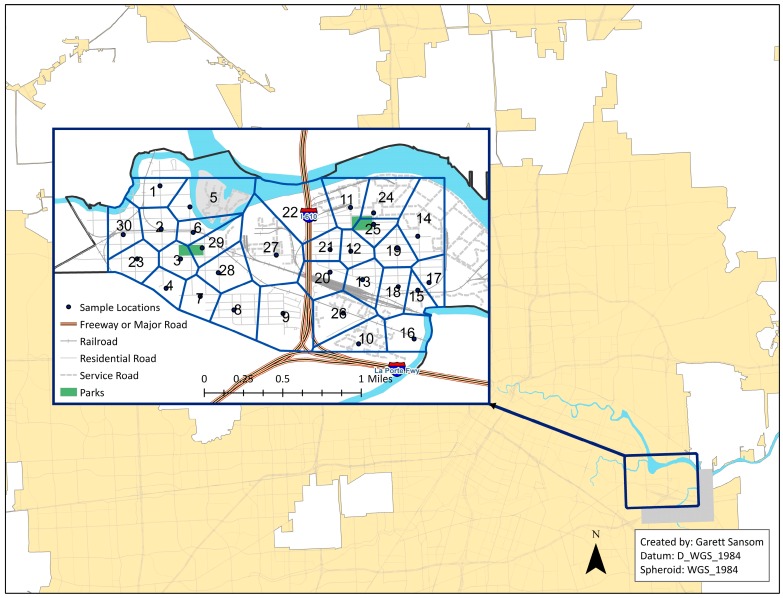
Water sampling locations within Thiessen polygon zones.

The community meeting with TEJAS and the Green Ambassadors allowed for local knowledge to help pinpoint locations that residents have noticed following rainfalls by offering the physical address or general location of problem areas. The neighborhood was partitioned into 30 separate clusters using the Thiessen polygon technique in ArcGIS (ESRI, Redlands, CA, USA) from the GPS locations and water sampling was conducted within each cluster (Figure 1).

Water sampling collection methods outlined by the US Environmental Protection Agency (EPA) Industrial Stormwater Monitoring and Sampling Guidelines [24] was utilized to ensure a quality sample collection procedure was established. The identified samples were collected from as near to the center of the pooled water as was feasible and acquired with a dip sampler that was replaced for each location. The collection team wore a new pair of nitrile gloves for each sample location to ensure no contamination occurred from handling the equipment. Samples were placed into 250 mL polypropylene laboratory containers with an HNO3 preservative and immediately placed into a Styrofoam cooler (Polar Tech, Genoa, IL, USA)

The samples were sent to A and B Labs, located in Houston, TX [25]. This lab is accredited through the National Environmental Laboratory Accreditation Program (NELAP) (T104704213-15-13). The lab provided data on the type and concentration of total metals (As, Ba, Cd, Cr, Pb, Se, Ag), in addition to mercury (Hg). When analyzing for As, Ba, Cd, Cr, Pb, Se, and Ag, US Environmental Protection Agency test method 200.7 was utilized for assessing trace metals in water. For mercury, EPA test method 245.1 was used, which is used for the determination of mercury in water by cold vapor atomic absorption spectrometry (CVAA). Quality control was assured through the use of laboratory blanks, laboratory control samples, and sample duplicates (LCS/LCSD), as well as a matrix spike and spike duplicate (MS/MSD) for all of the samples.

## 3. Results

### 3.1. Community Meeting

During the community meeting held in the Spring of 2015 with local citizens, as well as the advocacy and action groups TEJAS and the Green Ambassadors, the main interests surrounding public health were on the quality of the environmental conditions, human health impacts, and infrastructure. The abundance of large industrial trucks on residential roads was also mentioned. Drinking water, especially in the public schools, was thought to be far below the quality that they expected. Others mentioned the strong odor in the air and in the surface water, as well as that mosquitoes become quite severe certain times of the year. While certain efforts have been conducted already, such as air quality monitoring, there was concern that very little had been attempted to determine the quality of the environment in their neighborhood. Pooled surface water was thought to be especially polluted due to run off from industrial buildings surrounding the neighborhood. Concern over polluted water and flooding was mentioned on paper feedback forms as well as mentioned during verbal discussion which garnered broad agreement from those present.

### 3.2. Survey Results

Between 19 December and 26 December 2015, 109 (*N* = 109) surveys were collected with an overall response rate of 72.7%. Of the 192 homes that were approached contact was made with 150, this gave a contact rate of 78.1%. Of the respondents, 28.4% (*N* = 31) were completed by non-Hispanic white individuals, 62.4 percent (*N* = 68) Hispanic or Latino individuals, and 8.3% (*N* = 9) African American. Approximately half (49.5%; *N* = 54) were male and (50.5%; *N* = 55) were female. Race was coded as either Non-Hispanic White or Non-White to account for the relatively low amount of responses from African American participants (Table 1). Census data shows that within the neighborhood 87% are Hispanic, 10% identify as African-American, and only 2% are non-Hispanic white, significantly different than our survey results [18].

The survey results allowed for the identification of perceived community issues within their neighborhood (Table 2). On all issues, the majority of the community felt that the identified areas in the survey were a problem in their neighborhood. While waste facilitates and industrial buildings surround the residential areas of Manchester, there was a difference between the responses on whether it was a problem. Of the respondents 79.82% (*N* = 87) thought there were too many industrial buildings, while 68.81% (*N* = 75) thought waste facilitates were a problem. The survey also showed that standing water within the neighborhood was of concern, with 70.64% (*N* = 77) of respondents identifying it has a problem. Infrastructure was also identified as an issue of concern in two ways; 69.44% (*N* = 75) of respondents felt that too many homes in the neighborhood needed repairs and 69.72% (*N* = 76) claimed that road infrastructure, as dictated by potholes, was a problem in the community.

The strong agreement between all of the identified issues on the survey was also reflected with different gender and racial categories (Table 3). The agreement between these categories is strongest with concerns surrounding living near too many industrial buildings. A higher proportion of Non-Hispanic White individuals indicated that their community had a problem with waste facilities, standing water, poor infrastructure, and buildings that need repair compared to their counterparts. Of the Non-Hispanic White participants 83.87% (*N* = 31) of individuals claimed that their neighborhood had a problem with standing water, a higher proportion when compared to nonwhite respondents, where 65.39% (*N* = 78) thought standing water was a neighborhood problem.

While the quantitative results show the widespread agreement with environmental and community issues within the neighborhood, respondents provided remarks on various issues as well. In open ended comments, one respondent stated that they “smell gas inside (their) home”, and that there is “flooding around their area” while another pointed out that their “skin hurts when showering from chemicals”. Concerns over pollution, refineries, and industry were repeated by many respondents as areas of most concern, however there were also issues surrounded disillusionment with expectations of the future. One individual stated that “the government does not care about them because so many (are) black and hispanics” and another said “refineries don’t do anything for community”.

### 3.3. Surface Water Sampling

The results of the water quality sampling indicated that there were concentrations of barium in every location sampled, arsenic was present in eight locations, chromium in ten, lead in twelve, and mercury in two areas (Table 4). Many of the locations exceeded the levels set by the EPA with the national recommended water quality criteria for chronic exposure for aquatic life [26]. The levels of lead in the surface water samples showed a great amount of variety, and in one instance, levels were far above state and national levels. Of the twelve locations identified to contain lead, one of the samples had a level of 1448 (μg/L), and two other locations had levels exceeding 100 (μg/L). While mercury was only identified in two of the zones, each location had a concentration of 10 (μg/L). It is important to note that zone 4 had elevated concentrations of every found contaminant within the sampling criteria. While silver was tested for, no concentrations were high enough to allow for verification within this neighborhood.

## 4. Discussion

Our cross-sectional study was designed to evaluate if the concerns of local interest groups and residents who live in an area characterized by environmental justice issues and a high risk for natural disasters would be verified through environmental sampling and community surveys. The findings of this research suggest that not only did local citizens and interest groups understand the issues within their neighborhood, but that using the local knowledge already present within the community improved the quality of research by directing our research towards surface water assessment. Many of the issues raised by the community warrant future research projects, but the findings of the standing surface water add credence to the many other concerns expressed by those who live within the neighborhood.

The concerns expressed to the RCCCP specifically indicated a concern for the quality of the standing water, this concern was echoed across genders and racial composition in the survey responses, and this apprehension proved to be justified from the lab analyses. Several of these zones have issues with many heavy metals, specifically Zone 4 which had high levels of arsenic, barium, chromium, lead, and mercury. It should be noted that Zone 29, which had elevated levels of lead and chromium, as well as detectable amounts of barium and chromium, is in a public park heavily utilized by residents. The findings of this research will also help assess the overall community functioning to better understand the expected outcomes in the event of a natural disaster. Many of these findings can act as a mitigating or effect modifying factor during hazard events.

While these findings offer a troubling insight into the environmental conditions in the Manchester neighborhood, it also underscores the importance of utilizing local knowledge in every stage of environmental and public health research. The relatively high response rate to the survey, and identifying surface water as a concern, were highly influenced by the buy-in of community members. The initial locations of areas of surface water pooling were provided during the initial community meeting by offering physical addresses or locations based on landmarks such has parks and businesses to the research team. A community presentation is planned for the summer of 2016 to relay the findings of this research to the community as a whole, this presentation was requested and is being co-planned by TEJAS the RCCCP.

The benefit of utilizing community partnerships has been experienced with other investigative endeavors. Researchers Edgren et al. [27] of the Community Action against Asthma (CAAA) group in Detroit, MI trained and depended on community members to conduct household surveys that collected data relating to environmental conditions associated with asthma. The rapport created between respondents and interviewers was reported to be one that could not be duplicated by the research team themselves. Furthermore, the group Programa Para Responder an Emergencias con Preparación (PREP) is a community-based, participatory research program lead by researchers Eisenman et al. [28]. The PREP group found that engaging in participatory work helped inform and direct research and preparedness for the risk of natural hazards in a marginalized community. Our research continues to demonstrate the importance of community involvement.

This case study has several important limitations. This was a cross-sectional study and, therefore, only provides data on surface water conditions at a single point in time. Additionally, the environmental risk perceptions were collected at a single community meeting although these concerns have been well documented in a ship channel community assessment published by Air Alliance Houston, as well as research by the Texas Department of State Health Services [29,30]. The survey was also interviewer-administered; some research indicates that individuals tend to respond differently when speaking with an individual compared to self-administered surveys [31,32]. Despite the relatively high response rate, a small total amount of participants completed the survey, reducing our statistical power (*N* = 109). Non-Hispanic Whites were over-represented in our survey responses as compared to the U.S. Census data on race and ethnicity of Manchester residents [33]. Non-Hispanic Whites were more likely to complete the survey than their Non-White counterparts, which could cause selection bias within this study if Non-Whites’ concerns about the environment were substantively different than the Non-Hispanic White residents. Having the interview teams not approach homes that were completely fenced off or were deemed unsafe could have led to missing many residents with potentially different perceptions of environmental harm than those captured in this research. Providing additional opportunities for community participation, such as hosting further community meetings or offering surveys through the postal service, may have helped mitigate the disparity between our survey demographics and those provided by the U.S. Census. Future research attempts should utilize a more inclusive approach with several different opportunities for community members to participate.

## 5. Conclusions

While additional research is needed to assess the value and application of community engagement and participatory research, this study strongly suggests that using the ordinary knowledge of residents within local areas is highly valuable during every step of environmental and population research. Furthermore, these findings illustrate the environmental justice concerns that affect so many communities in the U.S. The environmental conditions within Manchester may be somewhat unique, but the experience of its resident’s likely echo those of other U.S. communities characterized by environmental justice issues.

## Figures and Tables

**Table 1:** Sample Characteristics.

Characteristics	*N* (%)
Gender	
Male	54 (49.5%)
Female	55 (50.5%)
Race	
Non-Hispanic White	31 (28.4%)
Hispanic or Latino	68 (62.4%)
African American	9 (8.3%)
Age in Years	
Mean (Standard Deviation)	45 (15.98)
Age in Groups	
<35	34 (31.5%)
36–50	28 (25.9%)
51–69	38 (35.2%)
70+	8 (7.4%)
Language	
Spanish	55 (50.5%)
English	54 (49.5%)

**Table 2:** Total number and percent of identified problems through surveying in the neighborhood of Manchester in Houston, TX in 2015 by issue.

Issue	*N*	%
Does your neighborhood have too many waste facilities	75	68.81
Does your neighborhood have too many industrial buildings	87	79.82
Does your neighborhood have flood related issues (standing water)	77	70.64
Do too many homes in your neighborhood need repair	75	69.44
Does your neighborhood have poor road infrastructure (potholes)	76	69.72

**Table 3:** Identified problems in neighborhood stratified by race, and gender.

Issue	*N*	*n* (%)
Too many waste facilities		
Non-Hispanic White	31	24(77.42)
Nonwhite	78	51(65.39)
Male	54	37(68.52)
Female	55	38(69.09)
Too many industrial buildings		
Non-Hispanic White	31	23(74.19)
Nonwhite	78	64(82.05)
Male	54	43(79.63)
Female	55	44(80.00)
Flood related (standing water)		
Non-Hispanic White	31	26(83.87)
Nonwhite	78	51(65.39)
Male	54	40(74.07)
Female	55	37(67.27)
Too many buildings that need repair		
Non-Hispanic White	31	24(77.42)
Nonwhite	77	51(66.23)
Male	53	36(67.93)
Female	55	39(70.91)
Poor road infrastructure (potholes)		
Non-Hispanic White	31	24(77.42)
Nonwhite	78	52(66.67)
Male	54	38(70.37)
Female	55	38(69.09)

**Table 4:** Heavy metal concentrations (μg/L) in 30 zones in the neighborhood of Manchester, TX.

**Zone**	**1**	**2**	**3**	**4**	**5**	**6**	**7**	**8**	**9**	**10**	**11**	**12**	**13**	**14**	**15**
Arsenic				180 *								38			11
Barium	60	85	544	3296	57	88	65	194	74	125	95	130	176	75	110
Chromium			46	363 *										11	
Lead		17 *	183 *	1448 *			17 *					34 *			
Mercury				10 *											
**Zone**	**16**	**17**	**18**	**19**	**20**	**21**	**22**	**23**	**24**	**25**	**26**	**27**	**28**	**29**	**30**
Arsenic	14		13		17				150 *	10					
Barium	274	88	452	153	176	135	299	731	136	46	132	180	55	209	940
Chromium	17		15		14		27	111 *						15	31
Lead	66 *		299 *		55 *		49 *	98 *						41 *	33 *
Mercury														10 *	

* Levels above National Recommended Water Quality Criteria for Chronic Exposure for Aquatic Life.

## Abbreviations

The following abbreviations are used in this manuscript:

RCCCP	Resilience and Climate Change Cooperative Project
EPA	United States Environmental Protection Agency
US	United States
CVAA	Cold Vapor Atomic Absorption Spectrometry
LCS	Laboratory Control Samples
LCSD	Laboratory Control Sample Duplicates
MS/MSD	Matrix Spike and Spike Duplicate
